# PLUNC Is a Novel Airway Surfactant Protein with Anti-Biofilm Activity

**DOI:** 10.1371/journal.pone.0009098

**Published:** 2010-02-09

**Authors:** Lokesh Gakhar, Jennifer A. Bartlett, Jon Penterman, Dario Mizrachi, Pradeep K. Singh, Rama K. Mallampalli, S. Ramaswamy, Paul B. McCray

**Affiliations:** 1 Department of Biochemistry and Protein Crystallography Facility, University of Iowa, Iowa City, Iowa, United States of America; 2 Department of Pediatrics, University of Iowa, Iowa City, Iowa, United States of America; 3 Departments of Microbiology and Medicine, University of Washington, Seattle, Washington, United States of America; 4 Department of Internal Medicine, The University of Texas Southwestern Medical Center, Dallas, Texas, United States of America; 5 Department of Internal Medicine, University of Iowa, Iowa City, Iowa, United States of America; Abramson Research Center, United States of America

## Abstract

**Background:**

The PLUNC (“Palate, lung, nasal epithelium clone”) protein is an abundant secretory product of epithelia present throughout the conducting airways of humans and other mammals, which is evolutionarily related to the lipid transfer/lipopolysaccharide binding protein (LT/LBP) family. Two members of this family - the bactericidal/permeability increasing protein (BPI) and the lipopolysaccharide binding protein (LBP) - are innate immune molecules with recognized roles in sensing and responding to Gram negative bacteria, leading many to propose that PLUNC may play a host defense role in the human airways.

**Methodology/Principal Findings:**

Based on its marked hydrophobicity, we hypothesized that PLUNC may be an airway surfactant. We found that purified recombinant human PLUNC greatly enhanced the ability of aqueous solutions to spread on a hydrophobic surface. Furthermore, we discovered that PLUNC significantly reduced surface tension at the air-liquid interface in aqueous solutions, indicating novel and biologically relevant surfactant properties. Of note, surface tensions achieved by adding PLUNC to solutions are very similar to measurements of the surface tension in tracheobronchial secretions from humans and animal models. Because surfactants of microbial origin can disperse matrix-encased bacterial clusters known as biofilms [1], we hypothesized that PLUNC may also have anti-biofilm activity. We found that, at a physiologically relevant concentration, PLUNC inhibited biofilm formation by the airway pathogen *Pseudomonas aeruginosa* in an *in vitro* model.

**Conclusions/Significance:**

Our data suggest that the PLUNC protein contributes to the surfactant properties of airway secretions, and that this activity may interfere with biofilm formation by an airway pathogen.

## Introduction

It was first recognized well over three decades ago that conducting airway secretions exhibit evidence of surface active material [2]. However, in contrast with alveolar surfactant [3], [4], an understanding of the nature, origin, and function of conducting airway surfactant has been elusive. Although it is generally agreed that the airway surface liquid of the large airways possess surfactant, as inferred from *in vivo* surface tension measurements made in large mammals [5], [6], [7], investigators have struggled to identify the factor(s) responsible for this reduction in surface tension. While upper airway secretions are known to contain a number of phospholipids associated with alveolar surfactant – most notably dipalmitoylphosphatidylcholine (DPPC) – the critical surfactant apoproteins surfactant protein B (SP-B) and surfactant protein C (SP-C) are absent from this region of the lung [5], [8]. This implies the presence of an analogous protein or proteins in the upper airways, capable of interacting with surfactant lipids to bring about surface tension lowering effects.

The volume and composition of the airway surface liquid in the conducting airways is maintained by the surface and submucosal gland epithelia, which secrete and absorb liquid and numerous factors involved in host defense and other processes. The PLUNC protein (Palate, Lung, Nasal Epithelial Clone; also known as SPLUNC1, LUNX, NASG or SPURT) is a proposed innate immune factor of approximately 25 kDa, which is present in human airway fluids including saliva [9], [10], nasal lavage fluid [11], [12], [13], [14], [15] and tracheal aspirates [16]. PLUNC is also present in secreted material from airway epithelial cultures [9], [16]. Immunohistochemical studies reveal strong PLUNC staining along the respiratory epithelium as well as in the tracheal submucosal glands [16]. Interestingly, it was recently reported that secreted PLUNC inhibits sodium transport by the epithelial sodium channel (ENaC) in air-liquid interface cultures of human airway epithelia [17], suggesting that PLUNC could play a role in airway surface liquid volume sensing and regulation.

The PLUNC gene is located within a gene cluster on human chromosome 20, which encodes at least 9 related PLUNC family members [18]. The PLUNC proteins are evolutionarily related to two serum glycoproteins, the phospholipid transfer protein (PLTP) and the cholesteryl ester transfer protein (CETP), as well as two innate immune factors, the bactericidal/permeability increasing protein (BPI) and the lipopolysaccharide binding protein (LBP). With the exception of CETP, whose gene lies on chromosome 16, the genes for PLTP, BPI and LBP reside on chromosome 20, near the 3′ end of the PLUNC gene cluster. All four proteins are thought to be structurally related to the PLUNC family members, based on a conserved exonic structure as well as computer modeling predictions [18]. Together with PLTP, CETP, BPI, and LBP, the PLUNC proteins form a superfamily known as the lipid transfer/lipopolysaccharide binding protein (LT/LBP) family, whose members share the ability to bind a variety of lipid substrates. Of note, BPI and LBP play well characterized roles in sensing and responding to Gram negative bacteria, mediated primarily through their ability to bind the Lipid A region of the bacterial cell wall component lipopolysaccharide [19]. In addition to lipopolysaccharide binding, the BPI protein is directly antimicrobial against Gram negative organisms [20]. Thus, the PLUNC protein's genomic context and its structural similarity to BPI and LBP have raised the possibility that PLUNC may possess a host defense function. However, work to date has failed to demonstrate potent, broad-spectrum antimicrobial activity by PLUNC.

Here we report that the PLUNC protein has surfactant activity, and that this activity may account for the low surface tension of airway secretions. We also present data to suggest a mechanism by which PLUNC may contribute to host defense. *In vitro* experiments with the opportunistic pathogen *P. aeruginosa* show that physiological concentrations of PLUNC inhibit the formation of biofilms, matrix-encased bacterial clusters implicated in the pathogenesis of many chronic infections. Biofilms persist in the face of intense immune responses and antibiotic treatment because physiologic changes inherent to clustered growth make cells far more resistant to killing than they are in the free-living (planktonic) state [21], [22]. Our results suggest that PLUNC could help prevent biofilm formation in human airways. This effect could maintain infecting bacteria in a susceptible state so they can be eradicated by other host defenses.

## Results

**Figure 1:**
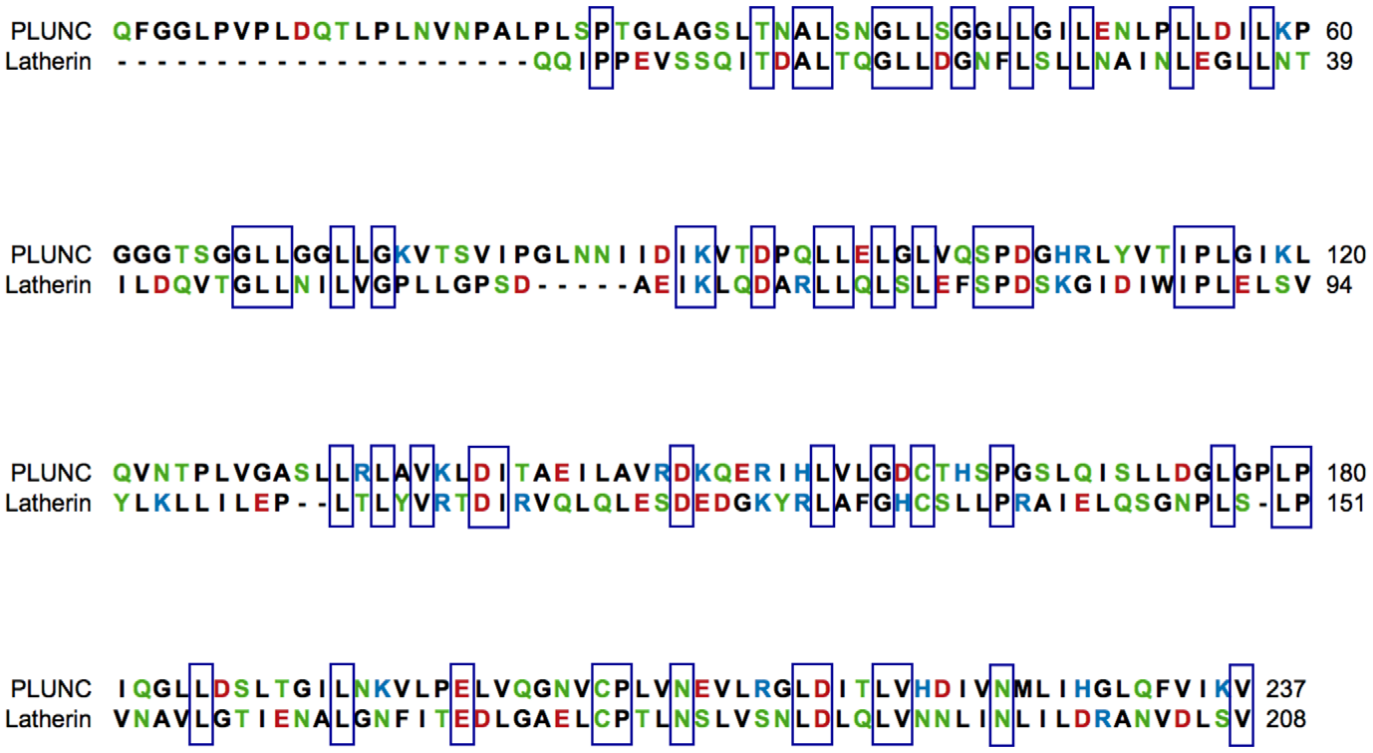
Multiple sequence alignment of PLUNC and latherin. Alignment depicts the amino acid sequence of human PLUNC (NP_057667), aligned with horse latherin (NP_001075328). The mature form of PLUNC was predicted using the SignalP 3.0 server (http://www.cbs.dtu.dk/services/SignalP/) to identify the likely location of the signal sequence [56]. Hydrophobic residues are shown in black, hydrophilic residues are green, basic residues are blue, and acidic residues are red. Blue boxes indicate conserved residues. Alignment was constructed using the CLC Bio Workbench 4.1.1 (CLC Bio A/S, Aarhus, Denmark).

Gene profiling studies performed previously indicated that PLUNC is among the most frequently represented transcripts in cDNA libraries constructed from human airway epithelia [23], suggesting that PLUNC protein is abundant in the conducting airways. To investigate the possible function of the PLUNC protein, we performed a BLAST analysis of human PLUNC against the non-redundant protein database at NCBI. This revealed a significant sequence similarity between PLUNC and the equine protein latherin (**Figure 1**), the only PLUNC homolog to have a described function to date. Latherin was originally isolated from horse sweat, and subsequently shown to display significant surface activity in a battery of *in vitro* assays [24], [25], [26]. On this basis, it was proposed that latherin is a surfactant protein. Like latherin, PLUNC is notably hydrophobic, with total hydrophobic residues making up approximately 44.7% of its amino acid sequence (**Table 1**). This is comparable to the observed overall hydrophobicity for latherin of 44.2%. For both PLUNC and latherin, this marked hydrophobicity is due in part to an extreme enrichment in the amino acid leucine, which makes up 23.2% of the total residues in PLUNC and 23.6% in latherin [26]. As shown in **Table 1**, latherin actually shares the greatest amino acid sequence identity with another member of the human PLUNC family, the breast cancer and salivary gland-expressed protein, or BASE, whose gene is the likely ortholog of latherin in the human genome [27]. However, due to a frameshift mutation in exon 6, it is thought unlikely that this gene codes for a functional protein product, and BASE is considered to be a pseudogene. Of all the human PLUNC family members, PLUNC stands out for its similarity to latherin in terms of both overall hydrophobicity as well as leucine content. This characteristic of both latherin and PLUNC suggests parallels with the well studied pulmonary surfactant proteins SP-B and SP-C. These proteins, which are proteolytically processed to give rise to short peptides representing their active forms, display overall hydrophobicities of 60.8% and 77.1% respectively (**Table 1**).

**Table 1:** Comparison of hydrophobicity between latherin and human PLUNC family members.

Protein	Length of mature protein (No. of residues)	Identity with latherin (%)	Total hydrophobic residues (%)	Leucine composition (%)	GRAVY
Latherin	208	–	44.2	23.6	0.330
hBASE	159	39	40.3	15.7	0.040
hPLUNC	237	28	44.7	23.2	0.563
hSPLUNC2	231	22	41.6	16.0	0.113
hSPLUNC3	233	22	38.2	9.9	−0.308
BPI	455	N/D	39.1	10.3	−0.075
LBP	456	N/D	40.4	13.8	−0.002
SP-B	79	N/A	60.8	17.7	1.027
SP-C	35	N/A	77.1	20.0	2.466

Amino acid sequences were obtained for horse latherin (NP_001075328) as well as the human LT/LBP family members BASE (AAO17728.1), PLUNC (also known as SPLUNC1) (NP_057667), SPLUNC2 (NP_542141), SPLUNC3 (NP_848561.2), BPI (NP_001716.2), and LBP (NP_004130.2). The analyses for SP-B and SP-C were based on the mature, proteolytically processed peptides for each, as described in [57]. To determine percent sequence identity with latherin, protein-protein BLAST (bl2seq) was performed to align latherin with each of the short PLUNC family members; for BPI and LBP, the sequence homology with latherin was so low that alignments could not be made (not done  =  “ND”). For the calculation of percent total hydrophobic residues, the amino acids isoleucine, valine, leucine, phenylalanine, cysteine, methionine, and alanine were considered to be the hydrophobic residues, based on the hydropathy index developed by Kyte and Doolittle [58]. The GRAVY (grand average of hydropathicity) value represents the sum of the hydropathy values for each amino acid in a protein, divided by the total number of residues. A protein with calculated GRAVY >0 is hydrophobic, while a GRAVY <0 indicates a hydrophilic protein.

**Figure 2:**
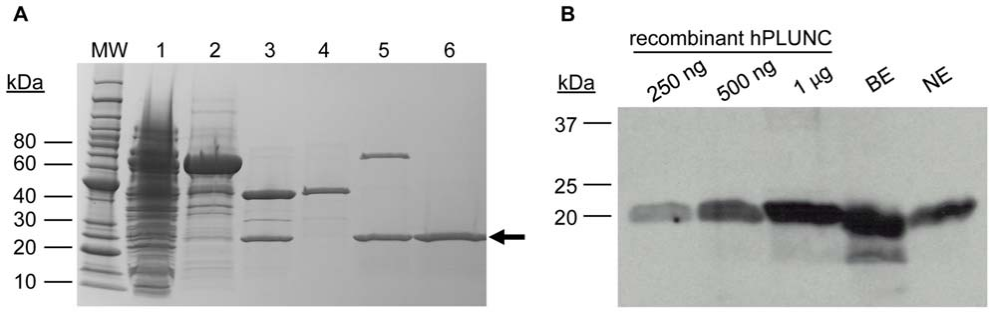
Expression and purification of recombinant human PLUNC. (A) Recombinant PLUNC protein was expressed in *E. coli* as described in the Methods. After centrifugation, the crude bacterial lysate (Lane 1) was passed over amylose resin and bound fusion protein was eluted from the resin using maltose. The recovered elution fraction (Lane 2) indicated a predominant product of approximately 69 kDa on a reducing SDS-PAGE gel, consistent with the predicted size of the MBP-PLUNC-6xHis fusion protein. Fusion protein was further purified by gel filtration, followed by a 16 hour cleavage with Factor Xa protease to remove the MBP tag. Factor Xa treatment gave rise to two cleavage products, MBP at 43 kDa and PLUNC-6xHis at approximately 26 kDa (Lane 3). After passing the cleavage products over nickel resin, MBP was observed in the flowthrough fraction (Lane 4), while the elution fractions contained PLUNC-6xHis and any remaining uncleaved His-tagged fusion protein (Lane 5). A final gel filtration step was used to separate PLUNC-6xHis from the uncleaved protein (Lane 6). Cleaved PLUNC-6xHis, in its final form, is indicated by the arrow. MW  =  molecular weight ladder (BenchMark Unstained Protein Standard; BioRad) (B) Representative immunoblot, in which increasing concentrations of purified recombinant PLUNC-6xHis were electrophoresed and probed using a monoclonal antibody directed against the human PLUNC protein, revealing a single immunoreactive band at the expected molecular weight in the range of 20–25 kDa. Immunoblotting also demonstrated the presence of native PLUNC protein in apical secretions from primary cultures of well-differentiated human bronchial epithelia (BE) and nasal epithelia (NE). See Methods for details.

Given this sequence similarity, we hypothesized that PLUNC is an airway surfactant. To test this idea, we first expressed recombinant PLUNC protein in *E. coli*, as a fusion protein containing a cleavable N-terminal maltose binding protein (MBP) tag as well as a C-terminal 6xHis tag for purification. The MBP tag served to increase solubility of the markedly hydrophobic PLUNC protein, as well as to provide an epitope that could be utilized for purification of the recombinant protein. As shown in Lane 6 of **Figure 2A**, we obtained full-length PLUNC at the expected size of approximately 26 kDa on a reducing SDS-PAGE gel, estimated to be ≥95% pure. The identity of this recombinant protein as PLUNC was verified by N-terminal sequencing and molecular weight determination using matrix assisted laser desorption/ionization - time of flight mass spectroscopy (MALDI-TOF) (not shown), as well as by immunoblotting with a polyclonal PLUNC antibody (**Figure 2B**). In keeping with the noted abundance of PLUNC transcripts in airway epithelial cDNA libraries, immunoblotting confirmed that human airway epithelial apical secretions are rich in PLUNC protein (**Figure 2B**); based on these and other immunoblotting studies, we estimate that native PLUNC protein is present in airway epithelial secretions at concentrations of approximately 10–250 µg/mL. We observed that recombinant PLUNC migrates very similarly to native PLUNC protein during SDS-PAGE.

**Figure 3:**
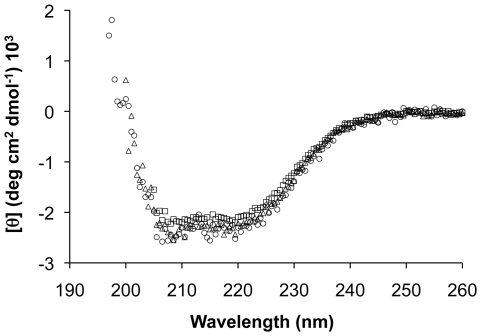
Circular dichroism spectra of recombinant human PLUNC. Circular dichroism spectra of recombinant human PLUNC. Molar ellipticities in the far-UV range (197–260 nm) are plotted for PLUNC at 0.54 mg/mL (circles), 1.08 mg/mL (triangles), and 2.16 mg/mL (boxes). Analysis of this data using the K2d server predicts that the secondary structure of PLUNC is 24-33% alpha-helical, ∼15% beta-sheet and 51–61% random coil.

Circular dichroism was used to investigate secondary structure of this recombinant PLUNC protein (**Figure 3**). The circular dichroism signal was negative in the 200–240 nm range, with dips around 208 and 222 nm indicating presence of secondary structure with some degree of alpha helicity. Analysis of the circular dichroism data using the K2d CD secondary structure server (http://www.embl-heidelberg.de/~andrade/k2d.html) [28] resulted in a prediction of 24–33% alpha-helical, about 15% beta-sheet and 51–61% random coil structure for PLUNC. In comparison, the N-terminal region of BPI, to which PLUNC is believed to be similar [18], is 27% alpha-helical, 46% beta-sheet and 27% random coil, while its C-terminal region (residues 250–456) is 24% alpha-helical, 44% beta-sheet and 32% random coil.

**Figure 4:**
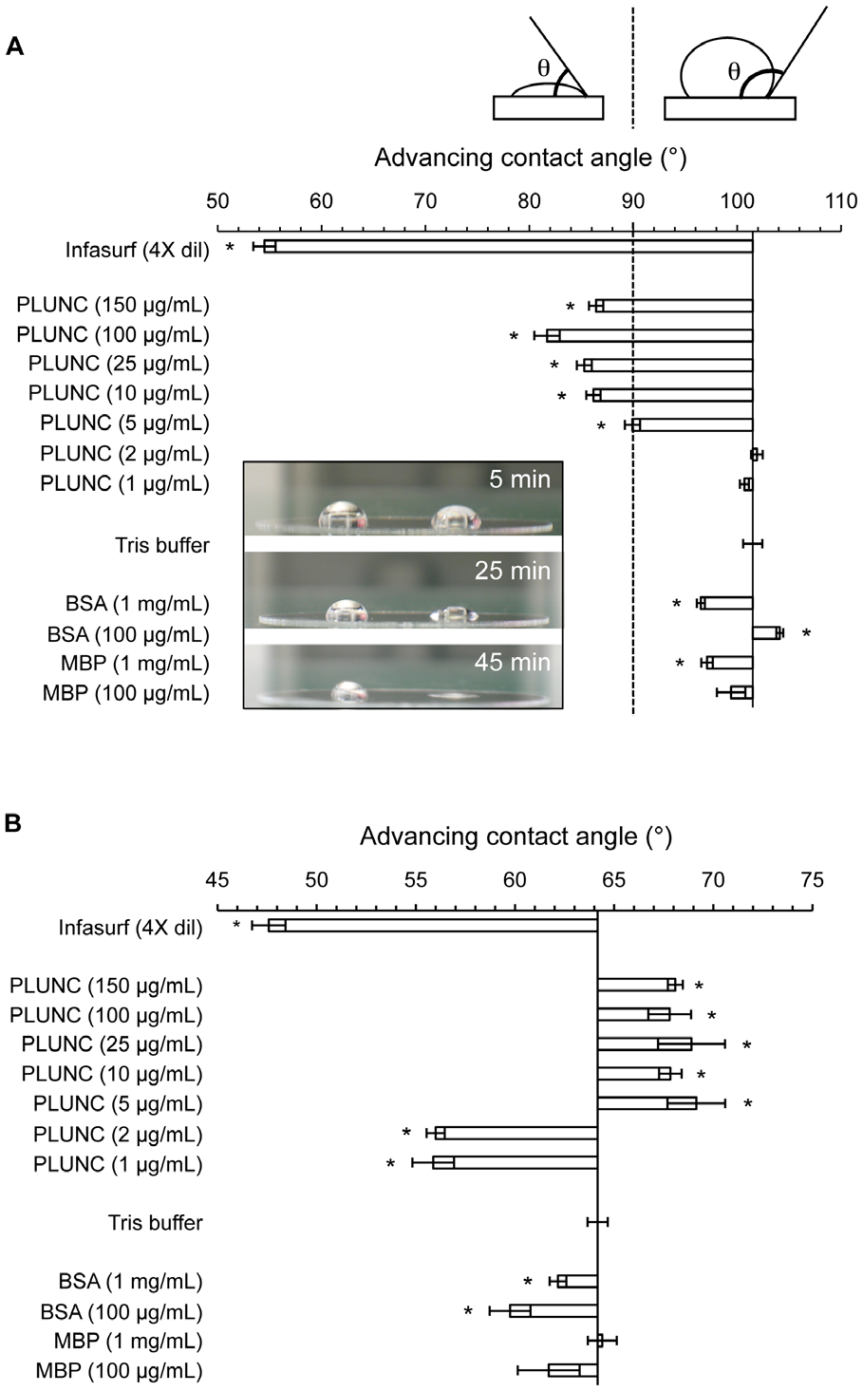
Contact angle measurements suggest that PLUNC possesses surface activity. (A) Advancing contact angles were measured by the sessile drop technique for various solutions dispensed onto siliconized glass, a hydrophobic surface. Bars depict the mean values for contact angles (in degrees) measured one minute after drops were dispensed onto the solid surface. Error bars represent the standard error about the mean (n = 6). Contact angles (θ) of less than 90° (dotted line in panel A) indicate wetting of the surface by the drop, whereas contact angles greater than 90° (to the right of the vertical line) indicate that a sample is “non-wetting”. The solid vertical line separates the solutions that have greater wetting ability than buffer alone (bars pointing left) from the ones that have lesser wetting ability (bars pointing right). Asterisks denote measurements that are significantly different from buffer, as determined by Student's two-tailed t-test (*P*-value <0.01). On a hydrophobic surface, PLUNC solutions transitioned from “non-wetting” to “wetting” at concentrations greater than 10 µg/mL. *Inset*: spreading behavior over time was compared for Tris buffer and a PLUNC-containing solution dispensed onto a hydrophobic surface. On each coverslip, the drop on the left is buffer, while drops of PLUNC (140 µg/mL) are shown on the right. Drop spreading was photographed after 5 minutes, 25 minutes, and 45 minutes, revealing that the presence of PLUNC conferred an increased tendency for an aqueous solution to spread on a hydrophobic surface. In panel (B), drops of test solutions were formed on unmodified glass, a hydrophilic surface, and advancing contact angles were measured as described above. On a hydrophilic surface, PLUNC enhanced wetting when at lower concentrations (1–2 µg/mL), while higher PLUNC concentrations (5–150 µg/mL) appeared to reduce wetting ability. Infasurf, a commercial lung surfactant, displayed significant wetting ability on both hydrophilic and hydrophobic surfaces.

To test PLUNC for surfactant activity, we first assessed the effects of this material on the surface activity of fluids at solid-liquid interfaces. As shown in **Figure 4**, we measured advancing contact angles for drops of various solutions on two types of surfaces. Samples were first studied on a hydrophobic surface (siliconized glass) (**Figure 4A**). In these experiments, the buffer control (20 mM Tris, 50 mM NaCl, pH 7.3) exhibited a mean contact angle of 101.5°±0.9. In contrast, a solution containing purified recombinant PLUNC at 150 µg/mL (the highest concentration tested) produced a statistically significant reduction of the contact angle to 86.4°±0.7, an effect that was diminished as the PLUNC concentration decreased. Contact angles observed for the control proteins BSA and MBP (both at the relatively high concentration of 1 mg/mL) were very similar to those for buffer, suggesting that the effects observed for PLUNC were relatively potent and specific to that protein.

In these studies, “non-wetting” (i.e., non-spreading) solutions, such as buffer, exhibited advancing contact angles of greater than 90°, while “wetting” solutions (such as Infasurf, a commercial pulmonary surfactant preparation extracted from bovine lung, containing surface active lipids and proteins) produced contact angles of less than 90°. Importantly, the presence of PLUNC in an aqueous solution appeared to cause contact angles to shift below this critical 90° angle, with PLUNC-containing solutions shifting from non-wetting to wetting behavior at concentrations between 5 and 10 µg/mL. The effects of PLUNC on surface spreading were particularly apparent in time course studies. As depicted in **Figure 4A** (inset), a drop of Tris buffered solution dispensed onto a hydrophobic surface exhibited very little surface spreading over a 45 minute time course, with some loss of volume due to evaporation. In contrast, a drop of a PLUNC-containing solution displayed nearly complete spreading over the same time period.

When solutions were dispensed onto a hydrophilic surface (glass) (**Figure 4B**), all solutions were observed to spread over the glass surface (as evidenced by contact angles of less than 90°). However, at PLUNC concentrations greater than 5 µg/mL, contact angles were increased with respect to the buffer control, suggesting that PLUNC at these concentrations interfered with the spreading behavior of the aqueous solutions. Below this 5 µg/mL concentration, the presence of PLUNC protein appeared to enhance the wetting ability of the solution.

**Figure 5:**
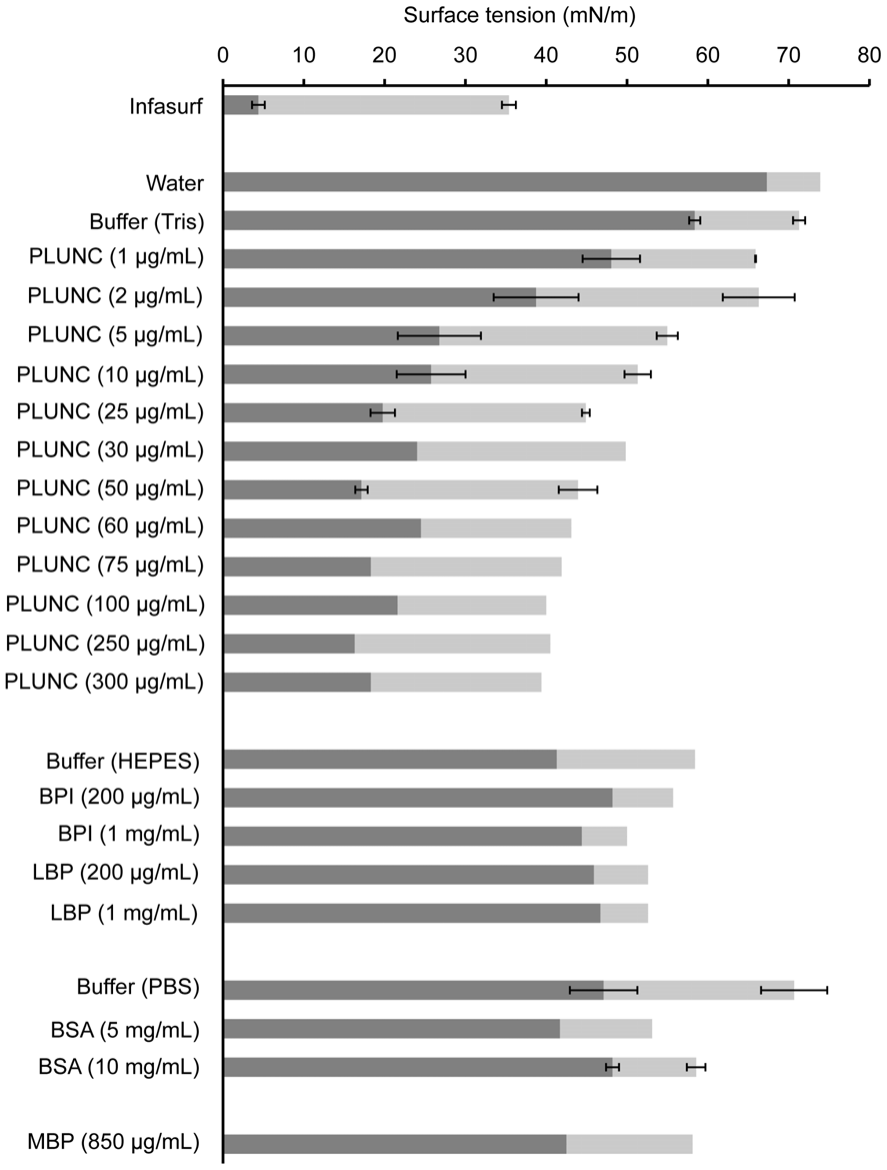
Reduction of surface tension at an air-liquid interface by PLUNC protein. The pulsating bubble surfactometer was used to measure dynamic surface tension in solutions containing increasing concentrations of recombinant PLUNC, as described in Methods. For each sample, the minimum surface achieved at 5 minutes of pulsation is shown as a gray bar. The range, representing the difference between the minimum and maximum surface tensions at the 5 minute time point, is depicted in white. Error bars, where present, represent the standard error about the mean (n = 3 experiments).

We next used a pulsating bubble surfactometer to measure the effects of PLUNC on surface tension at an air-liquid interface (summarized in **Figure 5**). We observed that PLUNC significantly and rapidly reduced the minimum surface tension of a Tris-buffered solution, to a value of approximately 18 mN/m. This effect was dose-dependent, with concentrations of only 5 µg/mL to 10 µg/mL required to observe maximal effects. Minimum surface tension could not be decreased beyond this value, no matter how much the PLUNC concentration was increased. As a positive control we also tested the effects of Infasurf, which contains a mixture of lipids and the surfactant proteins SP-B and SP-C. After 3–4 minutes of pulsation, minimum surface tensions of Infasurf preparations reached approximately 3 mN/m, consistent with potent surfactant activity.

**Figure 6:**
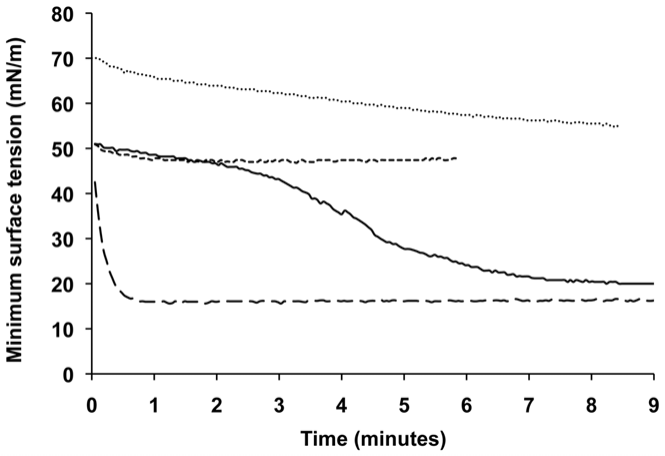
PLUNC interaction with albumin in the pulsating bubble surfactometer. The pulsating bubble surfactometer was used to measure dynamic surface tension in solutions containing mixtures of PLUNC and bovine serum albumin (BSA). Plotted values represent surface tension minima over time for Tris buffer (••••), BSA at 10 mg/mL (----), PLUNC at 10 µg/mL (― ―) or PLUNC (10 µg/mL) with BSA (10 mg/mL) (—). In the presence of BSA, it takes longer for PLUNC to reach its saturation value for minimum surface tension.

Interestingly, we found that the PLUNC relatives BPI and LBP had no significant effects on minimum surface tension even at concentrations as high as 1 mg/mL, suggesting that surface tension reduction is not a general property of all LT/LBP family members and is instead unique to the PLUNC protein. Similarly, we observed that highly concentrated solutions of BSA (5 mg/mL and 10 mg/mL) had only very modest effects on minimum surface tension. As the concentration of BSA used in these studies was orders of magnitude greater than the tested PLUNC concentrations, this strongly argues for a potent and specific surfactant activity by the PLUNC protein. We also examined the interaction between PLUNC and BSA, which, like many plasma proteins, has a well documented role in inhibiting pulmonary surfactant components [29]. In mixtures of PLUNC and BSA, BSA effects appeared to dominate early on in the surfactometer experiments, but these effects were always eventually overcome by the surface tension lowering activities of PLUNC (**Figure 6**). This pattern suggests that PLUNC effectively competes with BSA for space at the air-liquid interface.

A final parameter that was considered in the surfactometer studies was the range, in other words, the difference between maximum and minimum surface tension that was observed for each test solution during cycling. An effective biological surfactant will exhibit a large range, reflecting an ability to rapidly adjust surface tension throughout pulsation, while a poor surfactant will exhibit a relatively small range. As shown in **Figure 5**, not only did PLUNC display dose dependent surface tension lowering activity, but it exhibited ranges that were more similar to that for the known surfactant Infasurf than to those observed for control proteins including BSA and MBP.

The potency of the PLUNC protein's surface active properties raised the possibility that its surfactant activity may mediate its biological function in the airways. A clue to a potential function was suggested by work investigating mechanisms of biofilm detachment. Detachment is the process by which bacteria separate from biofilm clusters and return to the free-living (planktonic) state. In one established detachment mechanism, bacteria secrete surfactants that disrupt the biofilm structure, enabling individual bacteria to escape [1], [30]. These data, coupled with our finding that PLUNC has surfactant properties, led us to hypothesize that PLUNC might have anti-biofilm activity.

**Figure 7:**
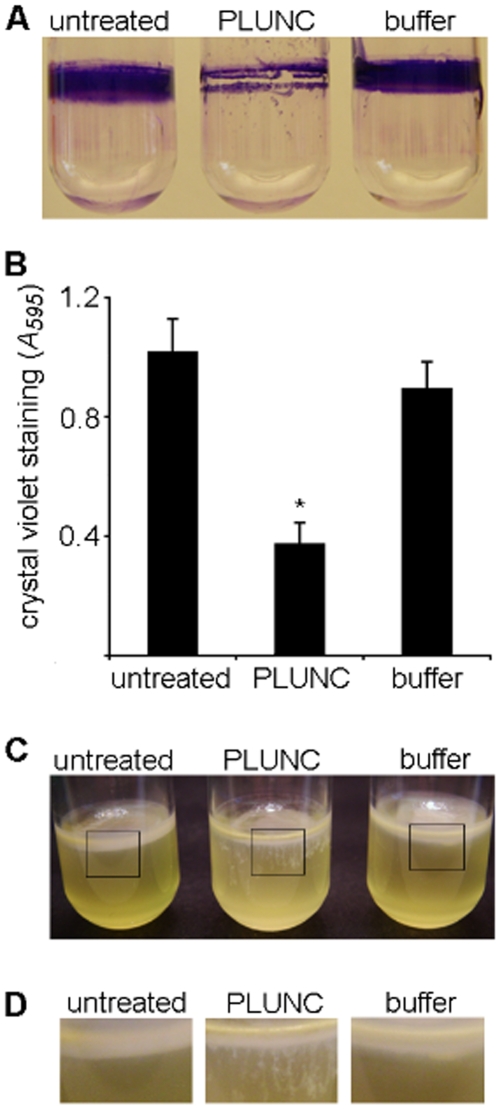
PLUNC inhibits biofilms growing at the air-surface interface. (A) Crystal violet staining of *P. aeruginosa* PA14 biofilms shows that PLUNC (100 µg/mL) reduced the biofilm biomass accumulating on the sides of culture tubes after 72 hours of growth. (B) The amount of crystal violet retained by biofilms was quantitated using optical density measurements. Growth of PLUNC-treated biofilms (n = 3) was significantly inhibited compared to non-treated and buffer-treated biofilms. Error bars show standard deviation. * *P<0.025.* (C) Biofilms formed at the air-liquid interface of unshaken cultures after 48 hours of growth. (D) Magnification of areas outlined in panel C shows that in media containing 100 µg/mL PLUNC, biomass spontaneously separates from the air-liquid interface biofilm and sinks toward the bottom of the culture tube.

We tested this hypothesis using the opportunistic pathogen *Pseudomonas aeruginosa*, a model organism for biofilm research. *P. aeruginosa* causes lethal chronic airway infections in patients with cystic fibrosis and bronchiectasis. Furthermore, the biofilm growth mode has been implicated in the pathogenesis of these and other chronic infections. Compared to the untreated and vehicle controls, PLUNC treatment interfered with biofilm growth at the air-liquid interface of static cultures. After 72 hours of growth, cultures treated with 100 µg/mL of PLUNC accumulated less biofilm biomass as assayed by visual inspection of crystal-violet stained biofilms (**Figure 7A**), and by measuring the amount of stain retained after repeated cycles of washing (**Figure 7B**). Similar results were obtained using 50 and 25 µg/mL of PLUNC (data not shown). In cultures grown with PLUNC, we also observed that biomass periodically separated from the biofilm and sank to the bottom of the culture tubes (**Figure 7C and D**). In contrast, biofilms that grew in control media remained as tight aggregates for the duration of the experiments (72 hours). The anti-biofilm effect of PLUNC was not due to bacterial growth inhibition, as PLUNC had no effect on *P. aeruginosa* growth [31]. These data show that PLUNC inhibits model *P. aeruginosa* biofilms *in vitro*, and suggest that it may contribute to anti-biofilm defenses *in vivo*.

## Discussion

Here, we describe for the first time a novel surface tension lowering activity for the PLUNC protein, a finding which may provide insight into whether and how PLUNC participates in host defense. Most PLUNC studies to date have focused on its proposed role as a host defense molecule, based largely on its weak primary sequence similarity to BPI and LBP. Given the noted abundance of the PLUNC protein in the nasal, oral and tracheal regions, it is often suggested that this protein may represent an innate immune molecule with properties uniquely suited to the milieu of the upper airways [18], [32], [33]. This is supported by the observation that PLUNC orthologs have thus far only been identified in the genomes of air-breathing mammals [27]. However, while it is often hypothesized that PLUNC acts as a directly antimicrobial protein, evidence for this is limited. Notably, PLUNC lacks the characteristic cationic charge density seen in many other antimicrobial proteins and peptides, including BPI. Bartlett et al. found no killing activity against *Escherichia coli*, *Pseudomonas aeruginosa*, or *Listeria monocytogenes* [31], while Chu and colleagues reported that recombinant murine PLUNC inhibited growth of *Mycoplasma pneumoniae* but inhibited *E.coli* only minimally (<15%) [34]. The lack of broad spectrum bactericidal activity suggests that PLUNC may target particular microorganisms, or that it contributes to host defense in unconventional ways. As discussed below, our findings indicate that one function of PLUNC may be to prevent chronic infections by inhibiting the formation of resistant bacterial biofilms.

The recognition that PLUNC is markedly hydrophobic, a property that it shares with the known surfactant protein and PLUNC family member latherin, initially led us to wonder whether PLUNC might have unusual effects on the behavior of aqueous solutions. We noted that the PLUNC protein is found *in vivo* in locations involving air-liquid interfaces, sites at which surface tension effects could be particularly relevant. Our contact angle analysis demonstrates that recombinant PLUNC significantly reduces the advancing contact angle observed for drops of aqueous solution on a hydrophobic surface, from 101.5°±0.9 for buffer alone to values between 80° and 90° for PLUNC at concentrations above 10 µg/mL. These results are comparable to those for latherin, which was observed to reduce the contact angle of a Tris-buffered solution to 86±1° at a concentration of 1 mg/mL (a nearly 10-fold higher concentration than the PLUNC concentrations used in our studies) [24]. These observations were further supported by pulsating bubble surfactometer experiments, which indicated that PLUNC reduces the surface tension of a Tris-buffered solution to as low as 18 mN/m. In terms of effectiveness as a surfactant, this places PLUNC somewhere between the activity that would be observed for a random, non-surfactant protein such as BSA - which has no or only a very modest effect on surface tension even at high concentrations - and Infasurf, a complex mixture of lipids and surfactant proteins (in particular, SP-B and SP-C) that can achieve surface tensions approaching 0 mN/m. Of note, SP-B and SP-C in their active forms exhibit a greater degree of hydrophobicity than do latherin and PLUNC (**Table 1**), which may explain in part the greater surface tension reducing potency of pulmonary surfactant compared to PLUNC in solution.

These surface tension effects were observed for PLUNC protein in the concentration range of 5 to 50 µg/mL. The concentration of PLUNC protein in airway surface liquid *in vivo* has not been clearly defined, although it is estimated that PLUNC makes up approximately 1% of the total protein in human nasal lavage fluid [15], and as much as 10% of the total protein in secretions from air-liquid interface cultures of human tracheobronchial epithelia [16]. As described in **Figure 2**, our studies suggest that PLUNC is present in airway surface liquid from well-differentiated human airway epithelia at a concentration of 10–250 µg/mL. Thus, we believe that these surface tension reducing effects by PLUNC occur in a concentration range that is physiologically relevant.

Several lines of evidence indicate that the conducting airways contain an agent or agents with surface activity. Measured surface tensions in upper airway fluids are significantly lower than would be expected in the absence of a surfactant. *In vivo* measurements of airway surface fluid lining the sheep bronchus indicate that surface tension in this region of the respiratory tract is 32±2 mN/m [7], while similar methods produced a surface tension of 31.9±0.5 for horse trachea [6]. In another study, surface tension was measured in porcine tracheal aspirates using a pulsating bubble surfactometer. In agreement with the *in vivo* findings, these tracheal aspirates exhibited minimum surface tensions in the range of 20–30 mN/m [5]. Similarly, Tarran and colleagues reported a surface tension of 33.9±0.6 mN/m for airway surface liquid at the apical surface of air-liquid interface cultures of human airway epithelia [35]. In contrast, the surface tension of pure water is 72 mN/m [36].

While it is generally believed that surfactant is present in the conducting airways, the nature and origin of this layer is not well characterized. Bernhard et al. [5] observed that surfactant extracts from porcine tracheal aspirates display a phospholipid composition similar to that recovered from bronchoalveolar lavage (BAL) fluid, suggesting an alveolar origin for these tracheal phospholipids. In this study, despite similar phospholipid compositions, surfactant extracted from tracheal aspirates exhibited a modest reduction in its surface activity with respect to BAL-derived surfactant. This raises the possibility that variable protein content might account for differences in surface activities between surfactant preparations derived from different regions of the lung. Interestingly, while surfactant extracted from tracheal aspirates was shown to be more proteinaceous overall than BAL-derived surfactant, this material exhibited significantly reduced levels of the pulmonary surfactant protein SP-A [5]. Furthermore, the hydrophobic surfactant proteins SP-B and SP-C were undetectable in surfactant of tracheal origin. These observations suggest that surfactant function in the conducting airways may involve additional proteins, which have yet to be defined.

We propose that PLUNC is one such protein. This is supported by the observation that purified recombinant PLUNC protein produces minimum surface tensions in a range that is consistent with observed *in vivo* surface tensions measured in the conducting airways of several species [5], [6], [7], [35]. Additionally, PLUNC belongs to a family of proteins whose common characteristic is the ability to bind lipids, suggesting that PLUNC may interact with lipids as well [18]. However, we emphasize that our contact angle and pulsating bubble surfactometer studies demonstrating surface active properties for PLUNC were all performed in the absence of any added lipids, suggesting that lipids are not necessary for PLUNC to exert its effects. PLUNC appears to possess surfactant-like properties on its own, in the absence of added lipids; in contrast, SP-B and SP-C reduce surface tension through their effects on the packing and compression of phospholipid layers. Thus, PLUNC may not be truly analogous to SP-B and SP-C. This question should be resolved by further investigation of lipid-binding activities by PLUNC. The broader question - determining the relative contribution of PLUNC protein to the overall surface tension of airway surface liquid in the upper airways - will require more in-depth studies involving genetic gain or loss of function approaches.

What purpose might surfactant serve in the large airways? In the distal lung, surfactant is critical to prevent the collapse of alveoli and the narrow passageways of the terminal bronchioles upon expiration [3]. However, the patency of the larger airways is maintained by elastic and cartilaginous support, so surfactant is unlikely to be critical in this region. It has been suggested that an airway surfactant may play a role related to lubrication; in support of this, there is evidence that surfactants can enhance rates of mucociliary transport [37], [38] and ciliary beat frequency [39]. Another possibility is offered by Schürch and colleagues, who suggest that low surface tension in airway secretions aids in the immersion of foreign particles into the airway surface liquid layer [7], [40]. Thus, it is possible that one role of PLUNC in the airways is to reduce surface tension to levels that enable immersion of inhaled bacteria and other particles into the airway surface liquid layer, facilitating their uptake by macrophages or elimination by other components of the innate immune system.

Our data suggest that the surfactant activity of PLUNC may provide protection against chronic infections by inhibiting the formation of bacterial biofilms. Conventional host defenses prevent infection by killing bacteria, inhibiting their growth, or facilitating their expulsion from sterile sites. Sometimes, however, bacteria escape initial killing. For example, during infections such as bronchitis, acute sinusitis, conjunctivitis, or infections associated with foreign bodies, bacteria can persist on mucosal surfaces for prolonged periods. If these bacteria form biofilms, immune responses, including those mediated by recruited phagocytes and adaptive immunity, are unlikely to be effective [22]. Host defenses that prevent biofilm formation could keep surviving bacteria in a susceptible state until robust immune responses are generated.

Precedents exist for anti-biofilm host defenses on mucosal surfaces. The protein lactoferrin, which is highly abundant in mucosal secretions (including airway secretions), has been shown to prevent biofilm formation by *P. aeruginosa* at concentrations below those that killed or prevented bacterial growth [41]. The iron-chelating activity of lactoferrin stimulates twitching, a specialized form of surface motility. This causes bacteria to wander over surfaces instead of forming cell clusters and biofilms [41]. The cationic host defense peptide LL-37 has also recently been shown to have specific anti-biofilm effects at concentrations below those that inhibited growth [42]. Our findings suggest that PLUNC may have similar effects, and raise the possibility that multiple anti-biofilm host defenses may exist. As is the case for antimicrobial factors that kill or inhibit bacterial growth, the presence of multiple factors could provide synergistic or redundant activity [43]. This could increase potency, widen the spectrum of organisms targeted, and perhaps prevent the emergence of strains resistant to anti-biofilm defenses.

The possibility that a host-derived surfactant disrupts biofilm formation is interesting in light of previous work showing similar activity of surfactants of microbial origin. As noted above, biofilm-forming organisms possess active mechanisms that allow them to leave biofilms and return to the planktonic state. Interestingly, *P. aeruginosa* uses the self-produced surfactant, rhamnolipid, to bring about biofilm detachment [1], [44] and rhamnolipids can also disrupt biofilms formed by *Bordetella bronchiseptica* [45]. Furthermore, *Pseudomonas putida* uses biosurfactants (Putisolvin I and II) for the same purpose [44], and surfactants can disrupt *Salmonella typhimurium* biofilms [46]. Recently, surfactants produced by *Lactobacillus acidophilus* were shown to inhibit *Staphylococcus aureus* and *Staphylococcus epidermidis* biofilms [47], raising the possibility that a surfactant-mediated anti-biofilm activity could be responsible for the probiotic effects of *Lactobacillus* species. Taken together, these findings suggest that surfactants may have broad spectrum anti-biofilm activity.

The notable surface active properties of PLUNC suggest that the molecule orients itself in a monolayer at the air-liquid interface, with the hydrophobic face of the molecule facing the air and hydrophilic regions in contact with the solution. Such a property is characteristic of surfactants. Although we currently have no structural data to formally demonstrate that PLUNC adopts such an amphipathic structure, we presume that such a phenomenon occurs. While the exact mechanism by which surfactants disrupt biofilms is not known, the presence of both hydrophobic and hydrophilic moieties may allow them to concentrate at physical interfaces and disrupt prevailing interactions [48]. This could dislocate surface adhesins that tether bacteria to each other, or to the biofilm matrix. Previous work shows that rhamnolipids can cause the release of LPS from *P. aeruginosa* [49] and they may have similar effects on other surface appendages. Another possibility is that surfactants disrupt and solubilize key matrix components, perhaps even incorporating them into micelles. The ability of surfactants to promote bacterial motility and alter cell surface charge [50], [51] could also help to release bacteria from biofilms. Whatever the mechanism, the anti-biofilm defense provided by PLUNC and other airway proteins could provide a fail-safe mechanism that prevents bacteria that survive initial defenses from assuming the intractable biofilm state. This activity could help explain the remarkable resistance of healthy mucosal surfaces to chronic infections.

In summary, we present evidence that PLUNC possesses surfactant-like properties, indicating that this protein plays a role in lowering surface tension in the upper airways. Furthermore, we show that PLUNC inhibits the formation of *P. aeruginosa* biofilms *in vitro*, suggesting that one biological function of the PLUNC protein may be to prevent chronic infections on mucosal surfaces. As such, these studies have revealed a novel and unexpected mechanism by which the PLUNC protein may participate in innate immunity in the conducting airways. Future work will be required to explore whether PLUNC inhibits biofilms formed by other bacterial species and to assess its overall contribution to host defense *in vivo*.

## Materials and Methods

### Ethics Statement

This study involved the use of human lung and nasal tissues obtained with written donor consent. Culture of human airway epithelia was carried out using methods approved by the Institutional Review Board of the University of Iowa.

### Expression and Purification of Recombinant PLUNC

The cDNA for human PLUNC (NCBI accession number NM_016583) was cloned into the plasmid vector pMAL-c2x (New England Biolabs). Full-length fusion protein, containing a cleavable N-terminal maltose binding protein (MBP) tag and a C-terminal 6xHis tag, was expressed in the *E. coli* strain BL21 Star (DE3) (Invitrogen Corp, Carlsbad, CA) and the protein purified by passing over amylose resin (New England Biolabs Inc., Ipswich, MA). MBP-tagged fusion protein was eluted from the amylose resin using Tris buffer (20 mM Tris, 50 mM NaCl, 0.001% Tween-20, pH 7.3) containing 10 mM maltose. Eluted fusion protein was concentrated to approximately 2 mg/mL under nitrogen pressure (40–50 psi) with stirring, using an Amicon Ultrafiltration membrane (30,000 MWCO; Millipore, Billerica, MA). Protein was then further resolved by passage through a Superdex 75 gel filtration column (Amersham; GE Healthcare, Pittsburg, PA). The resulting chromatogram indicated the presence of two peaks, corresponding to two distinct populations of recovered fusion protein. Both populations contained MBP-PLUNC-6xHis (confirmed by Coomassie staining of the fractions on SDS-PAGE gels); however, the higher molecular weight portion (represented by the first peak) appeared to be resistant to cleavage by Factor Xa protease (New England Biolabs, Inc., Ipswich, MA). Fractions corresponding to the second peak were pooled and concentrated to approximately 1 mg/mL using Amicon Ultra-15 centrifugal filter units (30,000 MWCO; Millipore, Billerica, MA), followed by removal of the MBP tag by Factor Xa cleavage carried out for approximately 16 hours at 4°C. The cleavage products were passed over nickel resin (Ni Sepharose 6 Fast Flow; GE Healthcare Biosciences Corp., Piscataway, NJ), and pure PLUNC-6xHis eluted from the resin using Tris buffer (20 mM Tris, 50 mM NaCl, pH 7.3) containing 500 mM imidazole. As a final purification step, the protein was again passed over a Superdex 75 gel filtration column and fractions containing pure (≥95%) PLUNC-6xHis were pooled and concentrated using Amicon Ultra-15 centrifugal filter units (10,000 MWCO; Millipore, Billerica, MA). After purification of the fusion protein and cleavage of the MBP tag, we observed a tendency for the remaining PLUNC product to precipitate; thus, 0.001% Tween-20 (v/v) was present at most stages in the purification process to improve the stability of PLUNC in solution. However, to prevent interference from Tween-20 in ensuing biophysical analyses of PLUNC, Tween-20 was removed from the buffers at the final purification step. Throughout the purification procedure, concentration estimates were calculated using absorbance at 280 nm (extinction coefficient 68,000 M^−1^ cm^−1^). Concentration estimates at the final purification step were obtained using the Bradford assay (Pierce Biotechnology, Inc., Rockford, IL).

### Culture of Human Airway Epithelia

Primary cultures of human airway epithelia were prepared from nasal tissue and bronchi by enzymatic dispersion using established methods [52]. Briefly, epithelial cells were dissociated and seeded onto collagen-coated, semipermeable membranes with a 0.4-µm pore size (Millicell-HA; surface area, 0.6 cm^2^; Millipore Corp., Bedford, MA). Cells were maintained in 2% Ultroser G medium at 37°C with 5% CO_2_. Twenty-four hours after seeding, the mucosal medium was removed and the cells were allowed to grow at the air-liquid interface. Only well-differentiated cultures (>2 weeks old) were used in these studies. The presence of tight junctions was confirmed by transepithelial resistance using a volt-ohm meter (World Precision Instruments, Sarasota, Fla.; resistance >500 Ω·cm^2^).

### Immunoblotting

Rabbit polyclonal PLUNC antiserum was generously provided by Dr. Philip Whitney at the University of Miami School of Medicine [16]. To collect secretions for immunoblotting experiments, the apical surfaces of polarized human airway epithelia were rinsed with PBS (containing Mg^2+^ and Ca^2+^) along with Complete Mini protease inhibitor (Roche Applied Science, Indianapolis, IN). Samples were collected in a total volume of ∼200 µL per 24-well plate and stored at 4°C until use. For SDS-PAGE, 22.5 µL of airway epithelial wash was loaded per lane. Samples were electrophoresed through 4–20% gradient Tris-HCl gels (BioRad Laboratories, Hercules, CA) and transferred to nitrocellulose membranes, followed by blocking overnight in TBS-Tween containing 5% powdered milk. Membranes were incubated with PLUNC antiserum, diluted 1∶1000 in TBS-Tween, for 1.5 hours. Following four 5 minute washes in TBS-Tween, membranes were probed with horseradish peroxidase (HRP) conjugated goat anti-rabbit secondary antibody (Sigma-Aldrich, St. Louis, MO) at 1∶20,000 for 1 hour. Another series of 5 TBS-Tween washes was performed, and bands were detected using SuperSignal West Pico Chemiluminescent Substrate (Pierce Biotechnology, Inc., Rockford, IL).

### Circular Dichroism

Circular dichroism spectra were recorded for recombinant PLUNC in the range 197–260 nm using an Aviv 62DS spectrometer (Aviv Associates Inc., Lakewood, NJ) and a quartz cuvette of 1 mm path length (Starna Cells, Atascadero, CA). Spectra were collected 5 times per sample and averaged for 3 different concentrations of PLUNC (0.54 mg/mL, 1.08 mg/mL and 2.16 mg/mL). Concentrations for the circular dichroism samples were estimated by Beer-Lambert's law using absorbance at 280 nm and a calculated extinction coefficient of 1490 M^−1^ cm^−1^. The percentage of secondary structure was calculated by deconvoluting the circular dichroism spectra using the online K2d CD secondary structure server, based on an unsupervised neural network algorithm [28], [53].

### Contact Angle Measurements

For wettability studies, 3–10 µL drops of solutions containing PLUNC, control proteins or Infasurf were dispensed onto Fisherbrand microscope glass cover slides (12-545-10, ThermoFisher Scientific, Pittsburgh, PA) or siliconized glass cover slides (Hampton Research, Aliso Viejo, CA). Advancing contact angles were measured by the sessile drop technique at room temperature, using a Ramé-Hart NRL 100-00 goniometer (Ramé-Hart Instrument Co., Mountain Lakes, NJ). For each sample, 3 replicates were performed, in 2 different spots on the coverslide, for a total of 6 measurements per sample. Contact angle measurements were collected at 1, 2 and 3 minutes after drop formation. Measurements for each time point were averaged to obtain the mean contact angle for each sample, as well as to calculate standard error about the mean. For all samples, contact angles were observed to decrease with time, suggesting possible evaporative effects as the experiment was not done in a controlled humidified chamber. Since the trends were identical for the samples at any given time point, only the 1 minute measurements are reported.

### Pulsating Bubble Surfactometer

Surface tension effects were measured using a pulsating bubble surfactometer (General Transco, Inc., Largo, FL), originally described by Enhorning [54]. To measure dynamic surface tension, a test solution was loaded into a disposable sample chamber, within which a spherical air bubble was formed that maintained contact with outside air. The air bubble was pulsated at a rate of 20 pulses per minute, between a defined minimum bubble radius of 0.4 mm and a maximum bubble radius of 0.55 mm (representing a 50% surface area change). Changes in pressure across the bubble interface were recorded and used to calculate surface tension values throughout cycling according to the Law of Laplace. For these studies, Infasurf® (Forest Pharmaceuticals, Inc., St. Louis, MO) was diluted 4-fold prior to loading into the surfactometer. Bovine serum albumin (BSA) for these studies was obtained from Sigma-Aldrich (St. Louis, MO) and maltose-binding protein (MBP) was obtained from New England Biolabs, Inc. (Ipswich, MA). Recombinant human BPI and LBP were generously provided by Dr. Jerrold Weiss at the University of Iowa. All proteins were maintained in a Tris buffer (20 mM Tris, 50 mM NaCl, pH 7.3), with the exception of BPI and LBP, which were maintained in HEPES buffer. Data were collected using the software supplied with the instrument and transferred to a computer for analysis. The minimum and maximum surface tension values for each pulsation cycle were extracted and plotted. To compare all samples simultaneously, surface tension values after 5 minutes of pulsation were used.

### Biofilm Culture Conditions and Growth Quantitation

Biofilms were cultured statically for 72 hours at room temperature in borosilicate glass (13×100 mm) tubes containing 1 mL of Luria-Bertani (LB) broth. Purified PLUNC was added to LB broth to give a final concentration of 100 µg/mL and a corresponding volume of buffer was added to LB broth for the vehicle control. Overnight cultures of *P. aeruginosa* PA14 were washed twice with phosphate buffered saline solution, and 30 µL were used to inoculate biofilms. Biofilm growth was quantified as previously described [55] except that methanol was used to elute the crystal violet stain adhering to the biofilm, after washing with PBS. Crystal violet staining was quantified by measuring the absorbance at 595 nm. Paired student's *t*-test was used to generate *P*-values.
